# Rapid Emergence of Multidrug Resistant, H58-Lineage *Salmonella* Typhi in Blantyre, Malawi

**DOI:** 10.1371/journal.pntd.0003748

**Published:** 2015-04-24

**Authors:** Nicholas A. Feasey, Katherine Gaskell, Vanessa Wong, Chisomo Msefula, George Selemani, Save Kumwenda, Theresa J. Allain, Jane Mallewa, Neil Kennedy, Aisleen Bennett, Joram O. Nyirongo, Patience A. Nyondo, Madalitso D. Zulu, Julian Parkhill, Gordon Dougan, Melita A. Gordon, Robert S. Heyderman

**Affiliations:** 1 Malawi Liverpool Wellcome Trust Clinical Research Programme, University of Malawi College of Medicine, Blantyre, Malawi; 2 Liverpool School of Tropical Medicine, Liverpool, United Kingdom; 3 Wellcome Trust Sanger Institute, Hinxton, United Kingdom; 4 University of Malawi College of Medicine, Blantyre, Malawi; 5 University of Malawi, The Polytechnic, Blantyre, Malawi; 6 Institute for Infection and Global Health, University of Liverpool, Liverpool, United Kingdom; University of Otago, NEW ZEALAND

## Abstract

**Introduction:**

Between 1998 and 2010, *S*. Typhi was an uncommon cause of bloodstream infection (BSI) in Blantyre, Malawi and it was usually susceptible to first-line antimicrobial therapy. In 2011 an increase in a multidrug resistant (MDR) strain was detected through routine bacteriological surveillance conducted at Queen Elizabeth Central Hospital (QECH).

**Methods:**

Longitudinal trends in culture-confirmed Typhoid admissions at QECH were described between 1998–2014. A retrospective review of patient cases notes was conducted, focusing on clinical presentation, prevalence of HIV and case-fatality. Isolates of *S*. Typhi were sequenced and the phylogeny of Typhoid in Blantyre was reconstructed and placed in a global context.

**Results:**

Between 1998–2010, there were a mean of 14 microbiological diagnoses of Typhoid/year at QECH, of which 6.8% were MDR. This increased to 67 in 2011 and 782 in 2014 at which time 97% were MDR. The disease predominantly affected children and young adults (median age 11 [IQR 6-21] in 2014). The prevalence of HIV in adult patients was 16.7% [8/48], similar to that of the general population (17.8%). Overall, the case fatality rate was 2.5% (3/94). Complications included anaemia, myocarditis, pneumonia and intestinal perforation. 112 isolates were sequenced and the phylogeny demonstrated the introduction and clonal expansion of the H58 lineage of *S*. Typhi.

**Conclusions:**

Since 2011, there has been a rapid increase in the incidence of multidrug resistant, H58-lineage Typhoid in Blantyre. This is one of a number of reports of the re-emergence of Typhoid in Southern and Eastern Africa. There is an urgent need to understand the reservoirs and transmission of disease and how to arrest this regional increase.

## Author Summary

Typhoid fever is a major cause of disease and death around the world, particularly in resource limited settings, although reports suggest that until recently it has been much less prominent in sub-Saharan Africa (SSA) than Asia. Estimates of the precise burden of this disease are, however, difficult, as diagnosis requires advanced laboratory diagnostics. This is a particular problem in much of SSA where long-term laboratory surveillance has been available in just a few settings. Queen Elizabeth Central Hospital (QECH), Blantyre, Malawi is one such setting; between 1998 and 2010, cases of Typhoid fever at QECH were both uncommon and responsive to all antibiotics. In 2011 a marked increase in highly antibiotic resistant Typhoid fever began, with 843 confirmed cases in 2013. A review of cases revealed that one in 40 patients died and one in five had complicated disease. A further study of the DNA of bacteria associated with the outbreak revealed a novel strain, common to Asia, has arrived in Malawi. This is one of a number of reports of the re-emergence of Typhoid fever in Southern and Eastern Africa. There is an urgent need to understand the reservoirs and transmission of disease and how to arrest this regional increase.

## Introduction

Typhoid fever, caused by *Salmonella enterica* serovar Typhi remains one of the most important infectious diseases globally, responsible for an estimated 26.9 million infections and 269,000 deaths in 2010 [1]. The accuracy of this estimate in sub-Saharan Africa (SSA) is limited by the paucity of diagnostic microbiological facilities in this setting, and the non-specific nature of Typhoid fever, which typically presents with non-focal sepsis, making syndromic diagnosis unreliable [2]. It has been suggested that the burden of Typhoid fever in Africa before 2010 has been over-estimated [3].

Whilst Typhoid has remained a major public health problem in Asia, there have been numerous reports consistently showing that nontyphoidal serovars of *Salmonella* (NTS) are a more prominent cause of bloodstream infection (BSI) in sub-Saharan Africa (SSA) [4]. In Blantyre, Malawi, there have been two well-documented epidemics of BSI caused by multidrug resistant (MDR) NTS serovars since 1998, while *S*. Typhi, until recently, has represented only 1% of *Salmonella* BSI [5].

The sequential acquisition of antimicrobial resistance (AMR) genes has been a prominent feature of Typhoid fever in Asia. MDR to amoxicillin, chloramphenicol and cotrimoxazole has been common since the 1980s [6]. This phenotype was largely associated with an IncH1 plasmid [7] and the use of any of these drugs will act to maintain this plasmid. This phenotype has led to the widespread use of fluoroquinolones in the management of Typhoid fever in Asia, the emergence and spread of diminished ciprofloxacin susceptibility and the increasing use of 3^rd^-generation cephalosporins and azithromycin there [8]. AMR surveillance data from SSA are sparse.

The Malawi Liverpool Wellcome Trust Clinical Research Programme (MLW) has conducted longitudinal surveillance of BSI in adult and paediatric medical patients presenting to Queen Elizabeth Central Hospital (QECH) with clinically suspected severe bacterial infection since 1998. In 2011 we detected an increase in microbiologically confirmed *S*. Typhi and here we report the emergence of a rapid and sustained increase in MDR Typhoid fever.

## Methods

### Setting

QECH is the largest government hospital in Malawi with 1,300 beds and provides free healthcare to Blantyre district (population approximately 1.3 million) and tertiary care to the Southern region of Malawi. MLW has conducted routine BSI surveillance since 1998 [9], obtaining blood for culture from all adult patients (age≥16) admitted to the medical wards with an axillary temperature over 37.5°C or with clinical suspicion of sepsis. In addition, blood cultures were obtained from febrile children (age<16) that were malaria slide negative or positive and critically ill, or from afebrile children with clinical suspicion of sepsis. Aerobic blood cultures were taken under aseptic conditions and before the administration of antibiotics. Sampling criteria did not change between 1998–2014.

### Microbiological methods

Automated blood culture has been undertaken at MLW using a standard aerobic bottle (BacT/Alert, bioMérieux Marcy-L'Etoile, France) since 2000, although prior to this, manual culture was undertaken [9]. The sample date, patient details (name, age and gender), blood culture result and antimicrobial susceptibility profile of any pathogen isolated were recorded. All isolates were identified using standard diagnostic techniques [10]. Salmonellae were identified by biochemical profile using API20E (bioMérieux Marcy-L'Etoile, France) and serotyped according to the White-Kauffmann-Le Minor scheme by antisera (Pro-Lab Diagnostics, UK) [11]. Coagulase-negative staphylococci, *Bacillus spp*., diptheroids and alpha-haemolytic streptococci other than *S*. *pneumoniae* (when there was no clinical suspicion of endocarditis) were considered as contaminants. MLW audits blood culture volumes and contamination rates [12].

Antimicrobial susceptibility testing was performed by disc diffusion using ampicillin, chloramphenicol, cotrimoxazole, cefpodoxime and ciprofloxacin discs according to British Society of Antimicrobial Chemotherapy methods and breakpoints, and isolates that were found to be resistant to ciprofloxacin or ceftriaxone by disc testing had e-tests (E-test macromethod, bioMérieux Marcy-L'Etoile, France) performed. Diminished ciprofloxacin susceptibility (DCS) was classified on the basis of a minimum inhibitory concentration (MIC) between 0.06–1 mg/L) [13]. Isolates were described as “fully susceptible” if susceptible to these five antimicrobials. MLW subscribes to the United Kingdom National External Quality Assessment Service (UK NEQAS) scheme. Isolates deemed clinically significant were frozen on beads at -80°C. Prior to October 2010, these data were entered into ledgers then double-entered into a validated database. Since then they have been directly entered into an electronic laboratory information management system (LIMS).

### Clinical management and data

Once the rapid increase in *S*. Typhi had been identified, a retrospective case note review of adults (aged ≥ 16 years) identified by *Salmonella* disease surveillance (College of Medicine Research Ethics Committee P.07/09/808) in the first 2 years of the increase (June 2011- June 2013) was undertaken. Clinical presentation, including focus of infection, HIV status (when known) and case-fatality rate at discharge were recorded. Data on survival at discharge amongst paediatric patients during the first 12 months of the increase was collected from the paediatric admissions ledgers. HIV testing was performed according to the Malawi national HIV rapid antibody testing protocol, using Determine (Alere, USA) HIV-1/2 tests as the first test in a serial testing algorithm. All positive test results were confirmed by Uni-Gold (Trinity Biotech, USA). First line treatment for uncomplicated Typhoid fever at QECH was oral ciprofloxacin for 7 days, at a dose of 750mg twice daily (bd) in adults and 20mg/kg bd (maximum 750mg) bd in children.

### Minimum incidence

Minimum incidence of Typhoid fever/100,000 patients/year was calculated using an estimated sensitivity of blood culture for the diagnosis of *S*. Typhi of 50%, therefore the number of positive blood cultures recorded in each year was doubled to provide a numerator. QECH serves the population of urban Blantyre, and the population size of Blantyre was estimated in the 1998 and 2008 census and population projections following the 2008 census have been published (National Statistics Office, Malawi: www.nsomalawi.mw/2008-population-and-housing-census.html). These numbers were used to provide a denominator.

### Genomic characterization of *S*. Typhi in Blantyre

In order to investigate whether this increase represented clonal expansion of a single lineage of *S*. Typhi, and to describe the full diversity of *S*. Typhi in Blantyre, blood culture isolates over the course of the surveillance period were selected for whole genome sequencing to represent the spectrum of antimicrobial susceptibility patterns in each year. These isolates were placed in a global context using previously sequenced *S*. Typhi isolates of known haplotype [14,15]. One *S*. Paratyphi (A270, Accession Number ERS223417) isolate was used as an out-group, to root the tree.

DNA extraction for whole genome sequencing was conducted on the Qiagen Universal Biorobot® (Limburg, Netherlands) using Qiagen All-for-one® extraction kits. Following DNA extraction, PCR libraries were prepared from 500ng of DNA as previously described [16]. Isolates were sequenced using Illumina HiSeq2500 machines (Illumina, San Diego, CA, USA) and 150 bp paired-end reads were generated.

Phylogeny was based on single nucleotide polymorphisms (SNPs) in conserved regions of the genome; WGS data for each of isolates was mapped to the reference *S*. Typhi CT18 [7] using SMALT (http://www.sanger.ac.uk/resources/software/smalt/: version 0.5.8). Phylogenetic modelling is based on the assumption of a single common ancestor, therefore variable regions, where horizontal genetic transfer occurs and repetitive regions, were excluded [15] [17]. A maximum likelihood phylogenetic tree was then built from the SNP alignments of the isolates using RAxML (version 7.0.4) [18]. The maximum-likelihood phylogeny was supported by 100 bootstrap pseudo-replicate analyses of the alignment data. The presence of plasmids was investigated using PlasmidFinder (Danish Technical University, Denmark: http://cge.cbs.dtu.dk/services/PlasmidFinder/).

### Ethics statement

Institutional approval for this study was obtained from the University of Malawi College of Medicine Research Ethics Committee (COMREC). All data analyzed were anonymised.

## Results

**Fig 1:**
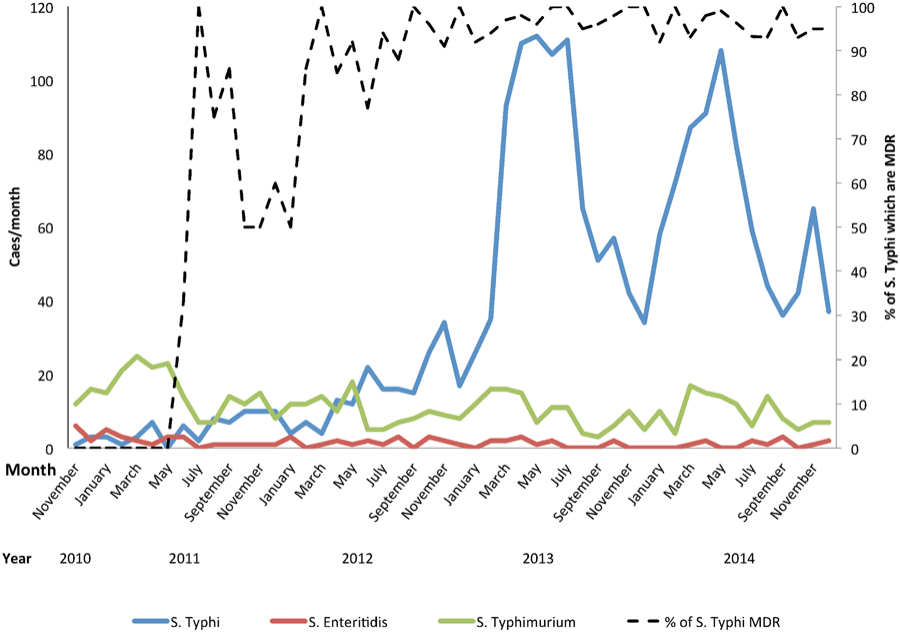
Monthly trends in bloodstream invasive *Salmonella* diagnosed at QECH from November 2010-October 2014.

Between 1998–2010, there were 176 microbiologically confirmed cases of *S*. Typhi at QECH in Blantyre, a mean of 14/year (Table 1); 12/176 (6.8%) were multidrug resistant (MDR) to ampicillin, chloramphenicol and cotrimoxazole and 147/176 (83.5%) were susceptible to all these commonly used antibiotics. All 176 were susceptible to both ciprofloxacin and ceftriaxone. A rapid increase in isolation of *S*. Typhi began in Blantyre in 2011, with 67 cases that year, 186 in 2012, 843 in 2013 and 782 in 2014. Although the total number of blood cultures taken has varied, the proportion of blood cultures yielding *S*. Typhi has risen from a long-term trend of ≤0.3% before 2011 to 5.7% of all blood cultures in 2014. The minimum incidence of Typhoid fever for urban Blantyre was estimated at 9.1/100,000 in 1998 and was stable until 2010 (1.4–9.1), but rose to 23.4/100.000 in 2011 and was 184/100,000 in 2014. A seasonal pattern has become apparent, with peaks of cases towards the end of the wet season and early in the dry season, when the prevalence of malnutrition is also highest (Fig 1).

**Table 1:** Temporal trends in *S*. Typhi isolation and antimicrobial resistance at QECH, Blantyre 1998–2013.

Year	Number of *S*. Typhi isolated	Number MDR*	Proportion MDR (%)	Number of Blood cultures	Proportion of blood cultures positive for *S*. Typhi	Estimated minimum incidence Typhoid fever/100,000
**1998**	24	0	0	8545	0.3	9.1
**1999**	13	0	0	7823	0.2	4.9
**2000**	5	0	0	8052	0.1	1.9
**2001**	4	2	50	7653	0.1	1.4
**2002**	4	0	0	8978	0.0	1.4
**2003**	9	0	0	11174	0.1	3.1
**2004**	17	0	0	10653	0.2	5.7
**2005**	19	0	0	12933	0.1	6.2
**2006**	18	0	0	10123	0.2	5.7
**2007**	10	4	40	9167	0.1	3.1
**2008**	16	0	0	8628	0.2	4.8
**2009**	19	0	0	7991	0.2	5.5
**2010**	18	6	33	8507	0.2	5.0
**2011**	67	42	63	9890	0.7	23.4
**2012**	186	168	90	10433	1.8	47.5
**2013**	843	823	98	12815	6.6	206.6
**2014**	782	754	97	13663	5.7	184.1

*MDR: Multidrug resistant to amoxicillin, chloramphenicol, cotrimoxazole

In 2014, 754/782 (97%) were MDR. One isolate with phenotypic DCS was detected in 2011(MIC 0.064mg/l), but none with outright ciprofloxacin resistance or ceftriaxone resistance have been detected as yet. There was greatest diversity of antimicrobial resistance patterns in 2010 and 2011 (Table 2); in 2010, the year before the increase began, 6/18 (33.3%) isolates were MDR and in 2011 42/67 (63.8%) were MDR (Table 3). To address the possibility that a greater diversity of strains was circulating in these years, isolates from these years were sampled most heavily for whole genome sequencing.

**Table 2:** Diversity of antimicrobial resistance phenotypes (n,%) amongst *S*. Typhi isolates from QECH, Blantyre 2010–2014.

	2010	2011	2012	2013	2014	Total
**Fully susceptible** †	8 (44.4)	18 (26.9)	11 (5.9)	11 (1.3)	15 (1.9)	63 (3.3)
**Cotrimoxazole**	2 (11.1)	1 (1.5)	1 (0.5)	2 (0.2)	1 (0.1)	7 (0.4)
**Chloramphenicol**	0 (0.0)	2 (3.0)	0 (0.0)	0 (0.0)	0 (0.0)	2 (0.1)
**Amoxicillin**	0 (0.0)	2 (3.0)	1 (0.5)	1 (0.1)	1 (0.1)	5 (0.3)
**Cotrimoxazole & Chloramphenicol**	0 (0.0)	0 (0.0)	1 (0.5)	1 (0.1)	0 (0.0)	2 (0.1)
**Amoxicillin & Cotrimoxazole**	1 (5.6)	0 (0.0)	3 (1.6)	5 (0.6)	7 (0.9)	16 (0.8)
**Amoxicillin & Chloramphenicol**	0 (0.0)	1 (1.5)	0 (0.0)	1 (0.1)	2 (0.3)	4 (0.2)
**MDR** *	6 (33.3)	42 (62.7)	168 (90.3)	823 (97.6)	754 (96.4)	1793 (94.7)
**Ciprofloxacin**	0 (0.0)	1 (1.5)	0 (0.0)	0 (0.0)	0 (0.0)	1 (0.1)
**Ceftriaxone**	0 (0.0)	0 (0.0)	0 (0.0)	0 (0.0)	0 (0.0)	0 (0.0)
**Total**	18	67	186	843	782	1893

^†^ Susceptible to amoxicillin, chloramphenicol, cotrimoxazole, ciprofloxacin, ceftriaxone.

*MDR: Multidrug resistant to amoxicillin, chloramphenicol, cotrimoxazole

**Table 3:** Phenotypic antimicrobial resistance patterns (n,%) of different clades.

Clade/Haplotype	1/H55	2/H50	3/H42	4/H52	5/H58
**Fully susceptible** †	6 (60.0)	3 (75.0)	0 (0.0)	10 (76.9)	6 (7.7)
**Cotrimoxazole**	0 (0.0)	0 (0.0)	0 (0.0)	2 (15.4)	0 (0.0)
**Chloramphenicol**	0 (0.0)	0 (0.0)	0 (0.0)	0 (0.0)	0 (0.0)
**Amoxicillin**	0 (0.0)	0 (0.0)	1 (100.0)	0 (0.0)	0 (0.0)
**Cotrimoxazole & Chloramphenicol**	0 (0.0)	0 (0.0)	0 (0.0)	0 (0.0)	0 (0.0)
**Amoxicillin & Cotrimoxazole**	0 (0.0)	0 (0.0)	0 (0.0)	0 (0.0)	3 (3.8)
**Amoxicillin & Chloramphenicol**	0 (0.0)	0 (0.0)	0 (0.0)	0 (0.0)	0 (0.0)
**MDR** * **number** (%)	4 (40.0)	1 (25.0)	0	1 (7.7)	69 (88.4)
**Total**	10	4	1	15	78

^†^ Susceptible to amoxicillin, chloramphenicol, cotrimoxazole, ciprofloxacin, ceftriaxone.

*MDR: Multidrug resistant to amoxicillin, chloramphenicol, cotrimoxazole

### Age distribution

**Fig 2:**
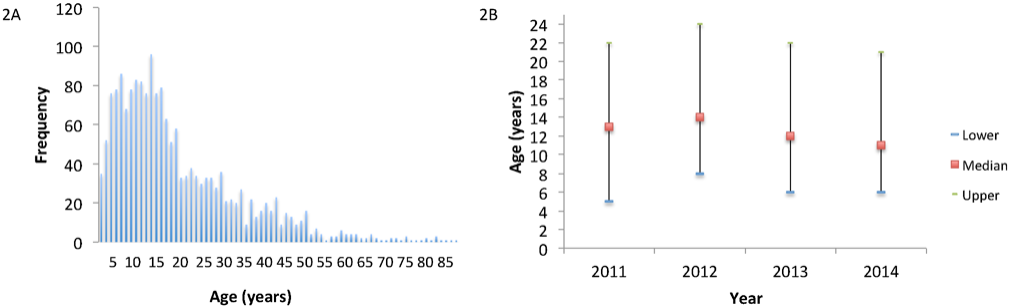
Age distribution of Typhoid in Blantyre 2011–14. 2A reflects the total age distribution frequency and 2B reflects the median age each year with interquartile range.

As is usual with Typhoid [6], the burden has fallen most heavily on children and young adults, with the majority of cases in children aged <16 years (1075/1693 [63%]). The age distribution since 2011 is depicted in Fig 2A. Of note, the median age (Fig 2B) has fallen from 14 (IQR 8–24) in 2012 to 12 (IQR 6–20) in 2014 (p = 0.01), as the increase stabilized.

### Retrospective case note review

A retrospective review of 77 adult admissions from the first 2 years of the increase was undertaken. 73/77 had typical presentations, consisting of fever without focus, or one or more of headache, severe sepsis or gastrointestinal symptoms. Although 5/77 (6.5%) were confused, none had any focal neurological syndromes similar to those reported in the outbreak in Neno, Malawi [19]. Only 1 patient (1.3%) had intestinal perforation. HIV status was known in 48 cases; 8/48 (16.7%) were HIV infected, which is comparable with the prevalence for the general population of Blantyre (17.8%), but is lower than for febrile adult admissions to QECH where 90% are HIV reactive [20]. 3/73 (4.2%) adult patients died.

A review of 330 paediatric admissions since the increase revealed 7 (2.1%) deaths. 212/248 (85.5%) children for whom fuller clinical details were available had a typical presentation with non-focal sepsis and a further 52/248 (21.0%) had disease complicated by one of the following; anaemia requiring transfusion (16 [6.5%]), meningism or decreased conscious level (13 [5.2%]), pneumonia (12/248 [4.8%]), intestinal perforation/peritonitis (9/248 [3.6%]), hepatitis (1/248 [0.4%]) and myocarditis (1/248 [0.4%]). All 7 deaths occurred in the group of patients presenting with complex disease including 4/9 intestinal perforation/peritonitis and the one patient with myocarditis. There were no cases of focal neurology or cranial nerve lesions [21]. HIV data were not available but the paediatric inpatient seroprevelence obtained through routine testing during this time was 13.6%.

Overall, the case fatality rate from culture confirmed Typhoid fever in adult and paediatric admissions to QECH following the increase in Typhoid fever was 2.5%.

### Clonal expansion of H58 *S*. Typhi

112 *S*. Typhi isolates from 2004–2013 were selected for whole genome sequencing to give the maximum temporal and antimicrobial susceptibility diversity of *S*. Typhi isolates contained within the MLW strain collection (see S1 Table for accession numbers). The years 2010 (18 isolates) and 2011 (67 isolates) showed greatest diversity of antimicrobial resistance profiles and a rise in the proportion which displayed the MDR phenotype, suggesting that these were the years in which a novel clade or clades were most likely to have become prominent in Blantyre. Proportionately more isolates were therefore sequenced from these years.

**Fig 3:**
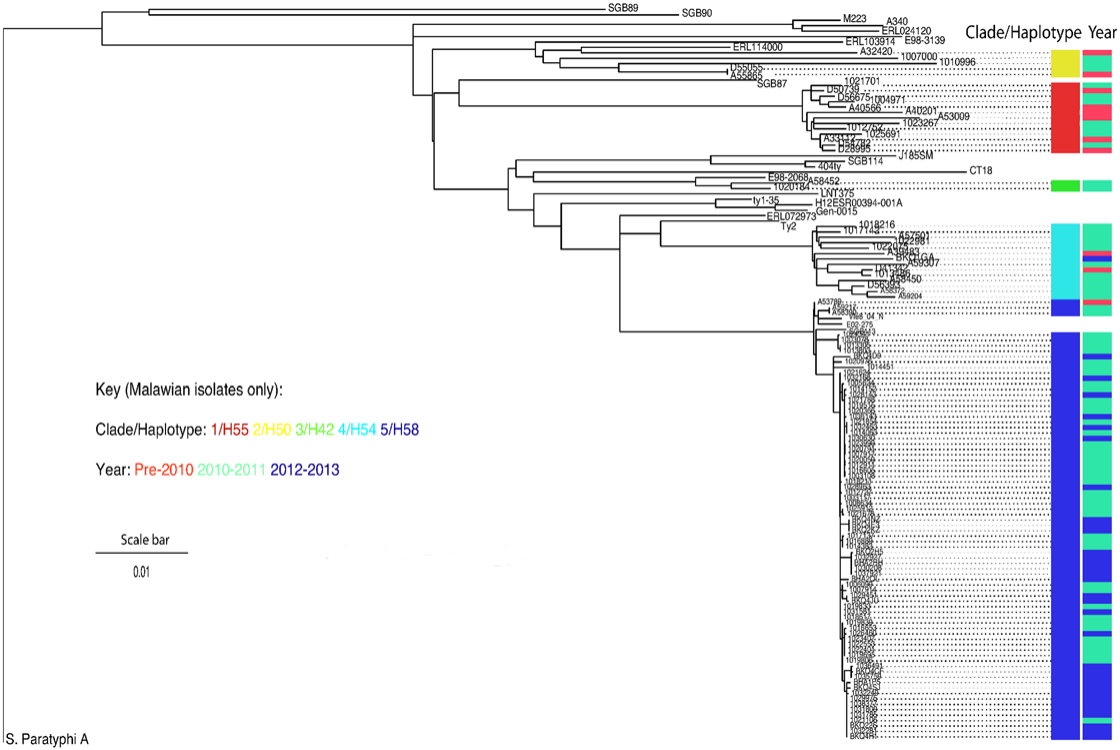
Maximum-likelihood tree of 112 isolates of *S*.Typhi from Malawi, placed in the context of 24 isolates representative of the global diversity of *S*. Typhi and highlighting the previous diversity of Typhi isolates and the recent clonal expansion of the H58 haplotype. The left column depicts lineage, the right column depicts time category. Scale bar reveals indicates substitutions/variable site.

**Fig 4:**
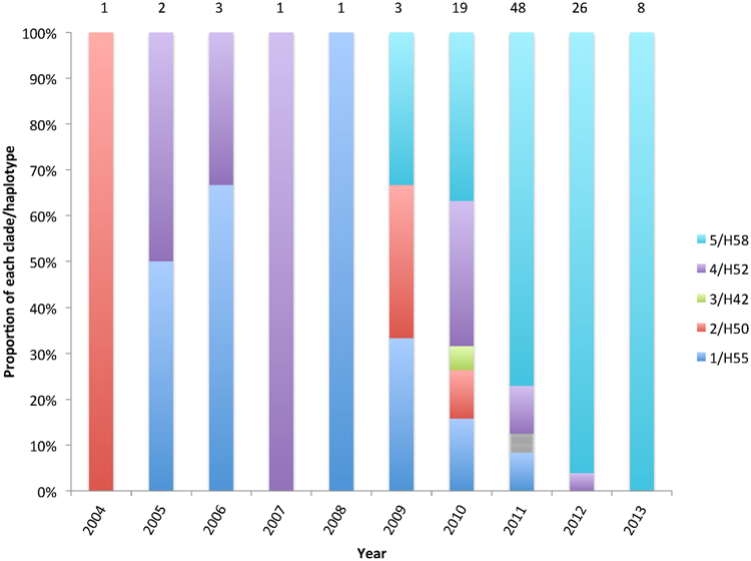
Proportion of sequenced *S*.Typhi isolates from MLW collection belonging to each haplotype, the number above each bar represents the total number sequenced for each year.

Maximum likelihood phylogeny of the 112 isolates placed in the context of a collection of *S*. Typhi isolates previously characterised by haplotyping revealed that five clades of *S*. Typhi were isolated from patients presenting to QECH, but that the recent increase was dominated by one clade, previously described as the “H58-haplotype”(Fig 3)[14]. H58 was first observed in this collection of isolates in 2009 (Fig 4), although this isolate was on a different branch to the majority of the rest of the H58 isolates. It suggests that, prior to 2011, the sporadic Typhoid diagnosed in Blantyre was caused by a diversity of *S*. Typhi clades, but that the H58-haplotype rapidly expanded in 2011. In this study, the H58 haplotype was much more strongly associated with MDR (89.3%) than the other types of *S*. Typhi circulating (21.4%). Plasmid finder and in-silico PCR were used to investigate the accessory genome of the H58 *S*. Typhi isolates. Both suggested that the IncH1 plasmid was not present in any of the H58-isolates. Review of the chromosomes of these isolates revealed that the MDR region carried on an IncH1 plasmid by reference strain CT18 has integrated into the chromosome of these isolates on a Tn21-like element between genes *cyaA* and *cyaY*. This has been observed previously in a Zambian epidemic [22].

## Discussion

Seventeen years of longitudinal surveillance data demonstrate that *S*. Typhi was an uncommon cause of BSI in adults and children in Blantyre, Malawi prior to 2011, and that an increase of MDR Typhoid fever, initially involving a wide range of clades but subsequently dominated by the H58-lineage began in 2011. This increase has now stabilized, but shows no sign of abating. This finding is consistent with a number of recent reports from Eastern and Southern Africa of outbreaks of Typhoid [21,23,24], which poses the questions, why is Typhoid emerging in Southern and Eastern Africa and how can it be controlled?

The rapid emergence of the H58-lineage of *S*. Typhi is likely to be associated with its spectrum of antimicrobial resistance. Chloramphenicol and amoxicillin are widely available in the community in Blantyre district and cotrimoxazole prophylaxis therapy is used as part of the ARV-programme. This clade is associated with resistance to all three agents and the widespread and poorly regulated use of these antimicrobials is likely to maintain this phenotype. Although *S*. Typhi and NTS appear to cause BSI in different patient groups, all 3 outbreaks of invasive *Salmonella* disease in Blantyre have shared this resistance pattern [5]. In contrast, DCS is a common feature of H58-*S*. Typhi in South Asia, therefore it is notable that DCS has yet to be reported in Blantyre. It is also important to note that since the increase, Typhoid fever has been associated with significant mortality and with complicated disease causing morbidity, despite the availability of fluoroquinolones for treatment, which have been available for treatment at QECH since 2002.

There have been two previously documented discrete epidemics of iNTS disease in Blantyre, the first caused by *Salmonella* Enteritidis (1999–2002), and the second by *S*. Typhimurium (2003–2010). iNTS disease has been associated with malaria, malnutrition and HIV [25], whereas malaria does not appear to be a risk factor for Typhoid [26] and HIV may be protective against Typhoid [27]. In Blantyre, there has been a rapid and successful rollout of antiretroviral therapy, including an increasingly effective prevention of mother to child transmission programme [20]. Whilst this study does not provide evidence that HIV is protective against Typhoid fever, there is no suggestion that it is a predisposing factor as is the case for NTS. There has also been an expansion of malaria control interventions. Both of these interventions are predicted to reduce the proportion of the population that is susceptible to iNTS disease. It is also possible that the previous *S*. Enteritidis epidemic, which like S. Typhi is a member of *Salmonella* serogroup D, led to heterotypic immunity to Typhoid fever in the population of Blantyre, which 10 years after the end of the epidemic is now in decline and this possibility should be explored further. However, it is also possible that the arrival of the H58 MDR-associated haplotype that has been broadly reported in Asia and locally in Africa has in itself been a driving factor in this rapid increase in disease. Again, further work is required to determine this.

Typhoid control has previously been associated with improvements in drinking- water quality, sanitation and hygiene practices [6,28–30]. This outbreak has occurred following an increase in improved water source coverage in Malawi, however there are reports that many of these water sources may have become contaminated and river water pollution in the city has become a problem [31]. Use of inadequately treated sewage water and human faecal manure from ecological sanitation latrines for growing vegetables must also be considered as potential contributors to this increase [32]. A recent survey of the residents of informal settlements in Blantyre found that only 7% of respondents practiced hand hygiene after defecation [33]. These pressures have been compounded by a dramatic increase in population growth; the population of Malawi has been growing at an estimated 2.8%/year based on national census projections, and Malawi is also urbanizing at the rate of 3% p.a. [34]. These increases in urban population density without proportionate improvement in access to water and sanitary facilities may have played a role in facilitating this outbreak.

We report microbiological data from a single center in Blantyre, however QECH is the sole free-of-charge hospital in Blantyre, and this finding is likely to be generalizable to the rest of urban Malawi. The clinical data are retrospective, based on available case-notes. Although the DCS phenotype was not detected, the isolates were not tested against nalidixic acid, which is the most sensitive method of detecting emerging DCS by disc testing [35]. It was only possible to sequence a selection of isolates from the MLW archive and we have sampled fewer isolates from low-incidence years, so we cannot be sure precisely when H58-S. Typhi first arrived in Blantyre.

There has been rapid emergence of Typhoid in Blantyre not associated with HIV infection, initially involving a wide range of clades, but dominated by the MDR H58-lineage of *S*. Typhi. This is one of an increasing number of reports of outbreaks of Typhoid ever from the region. There is a critical need for a comprehensive description of the clinical and molecular epidemiology of this neglected tropical disease across Africa, in order to understand its true burden, to model its transmission dynamics and to inform vaccination trials.

## Supporting Information

S1 Table Isolates sequenced and their related accession numbers. (CSV)
